# Professional mental health aid for children, adolescents, and young adults during COVID-19 in Flanders, Belgium

**DOI:** 10.3389/fpubh.2025.1527141

**Published:** 2025-07-15

**Authors:** Emilie Muysewinkel, Lara Vesentini, Helena Van Deynse, Stephanie Vanclooster, Kenniscentrum Kinderrechten, Sofie Devarwaere, Johan Bilsen, Roel Van Overmeire

**Affiliations:** ^1^Mental Health and Wellbeing Research Group, Vrije Universiteit Brussel, Laarbeeklaan, Belgium; ^2^Department of Public Health, Vrije Universiteit Brussel, Laarbeeklaan, Belgium; ^3^Kenniscentrum Kinderrechten, Ghent, Belgium; ^4^Awel, Schaarbeek, Belgium

**Keywords:** mental health services, mental health, COVID-19, youth, stigma

## Abstract

Few studies have investigated whether children, adolescents, and young adults (aged 10–25 years) had access to professional mental health aid when they desired it during the coronavirus disease 2019 (COVID-19) pandemic. An online survey was conducted in Flanders, Belgium, to examine professional mental health aid access (PMHAA). In total, 982 respondents were included, of whom the majority were between 13 and 15 years old (44.8%) and female (81.4%). Of these, 64.8% felt a need for PMHAA. Of those wanting PMHAA, only 29.6% had an appointment with a mental health professional. Those who did not have PMHAA mostly did not do so because of fear of stigma (54%), not knowing where to search for professional mental health aid (38.2%), and finding the aid to be too expensive (12.9%). This study’s findings indicate a large unmet need for professional mental health aid during the COVID-19 pandemic among people aged between 10 and 25 years old. There is a need for a campaign to reduce mental health stigma in this population.

## 1 Introduction

During the COVID-19 pandemic, several government measures were taken to stop the spread of the virus. In many countries, social distancing was introduced, and work shifted from physical locations to online settings. Young people had to attend their classes online, as schools were closed. Although the mortality caused by the virus decreased over time, the COVID-19 measures also seemingly had other consequences. Studies have demonstrated that children, adolescents, and young adults experienced more issues with their wellbeing and mental health than before the start of the pandemic (1–6). This encompassed, among other things, an increase in depressive, stress, or anxiety symptoms compared to before COVID-19 (1–3, 7). Furthermore, studies have indicated that the likelihood of child abuse and maltreatment increased during the pandemic (8–11). Regarding suicidal ideation and behavior, studies provide conflicting evidence: some find higher rates of suicide and suicidal ideation among children, adolescents, and young adults, while others report the opposite (12, 13).

Consequently, there was an increased need for professional mental health aid access (PMHAA). However, due to COVID-19 restrictions, there were fewer possibilities to obtain such access (14–17). This was, as was the case in schools, professional mental health aid was limited as much as possible in physical form. The provision of professional mental health aid often shifted to virtual settings, which likely hindered some individuals from PMHAA (e.g., due to a lack of internet access) (18–20). Therefore, unsurprisingly, several studies have demonstrated unmet health needs among children, adolescents, and young adults during COVID-19 (21–24).

In the context of Flanders, it remains somewhat unclear whether there were unmet mental health aid needs or not, and if so, what the magnitude of this problem is. One study indicated that adolescents and young adults in Belgium generally had less access to services (21). However, the minimum age in that study was 15 years old and thus did not provide information for those younger than 15. Furthermore, the reasons for the lack of mental health access often remained unclear and understudied. Studies on PMHAA remain important to find out how mental health literacy and knowledge can be improved among young adults, as this remains a sorely understudied topic (25).

This study investigates access to professional mental health aid (PMHHA) specifically among children, adolescents, and young adults (between 10 and 25 years old) during the COVID-19 pandemic in Flanders. There is a strong need for such studies because this is a vulnerable group that is at risk of developing mental health issues (7).

## 2 Method

### 2.1 Population and design

In 2021, the population in Flanders that was between 10 and 25 years old was 1,084,135 (26). Approximately 16.3% had some form of mental health disorder (27). An online cross-sectional survey was developed in cooperation with Awel (a youth help line in Flanders) and Kenniscentrum Kinderrechten (in English: “Children’s Rights Knowledge Centre”), an organization that strives to strengthen and acknowledge the position of children in society. Central to this approach is the respect for and guarantee of their rights. Data collection occurred from 2 February to 2 March 2021. During this period, COVID-19 measures were still strict, with meetings with other people only allowed up to 10 people and only outside, and physical education was still not allowed. Cookies were used in the online survey to prevent multiple answers from the same individual.

The survey was developed in several phases: First, questions were evaluated with employees of Awel and WAT WAT, together with a pilot group of several adolescents and children, to verify whether questions were understandable for children, adolescents, and young adults. Then, adjustments were made to questions according to their feedback, such as the reduction of the number of questionnaire items, removing as many Likert scales as possible, and adding multiple-choice questions. Afterward, a copywriter of Ambrassade, another website targeting youth, simplified questions that were too complex in formulation. All questions were only available in Dutch. This was done as the partners who agreed to spread the survey were all Flemish organizations, and thus only provided information to youth in Dutch.

The link to the survey was put on several websites of several organizations for the wellbeing of adolescents and young adults in Flanders, such as Awel, Kenniscentrum Kinderrechten, WAT WAT, and Ambrassade. Awel is a Flemish organization that aims at low-threshold online conversations with youth about any topic, ranging from school, friendship, to mental health issues. During 2021, when data collection occurred, Awel had 28,886 contacts with youth, which was 2% more than in 2020 (28). Kenniscentrum Kinderrechten, WAT WAT, and Ambrassade are websites where young adults can search for information about various themes/subjects, including rights, mental health issues, and sexuality. Kenniscentrum Kinderrechten does not have children and youth as its primary target group. They mainly focus on practice, policy, and research. People who work for, by, or about children and young people. Through their website, they reach these people and, in a secondary way, also children, young people, and young adults themselves.

### 2.2 Measures

**Figure 1:**
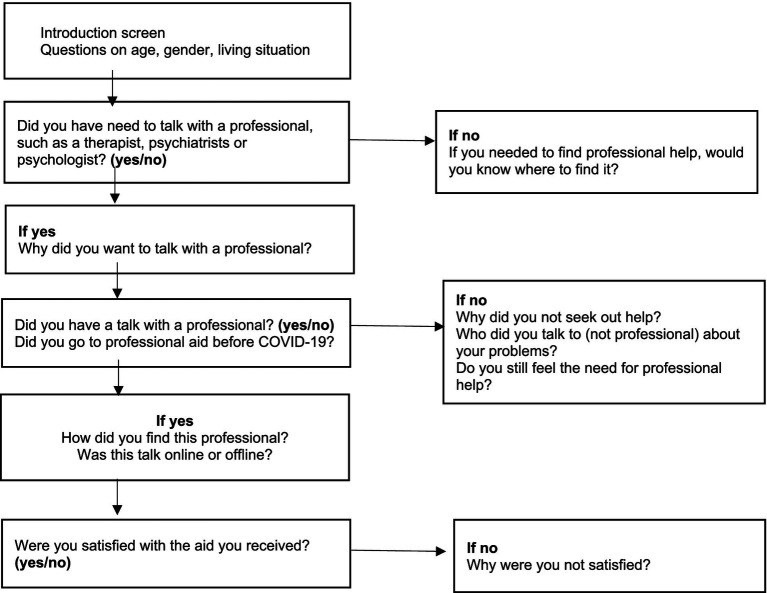
Scheme of measurements.

The survey had a complex structure as there were many different pathways to answering it, as illustrated in Figure 1. Collected demographics were age, gender, and living situation. Age categories included 7–9, 10–12, 13–15, 16–17, 18–19, 20–25, and 25+. Living situation was categorized as “living with one parent at a time (e.g., parents are divorced),” “I live in the same house as my parents,” “I live in another family than with my parents (e.g., foster parents, grandparents,…),” “I live in a group (e.g., hospital, asylum centre…),” “I live alone (e.g., student home),” and “I live in another way (free text field).” Gender had options “boy,” “girl,” “other,” or “no answer.” Several questions were only answerable with “yes” or “no,” especially those concerning PMHAA: “Did you have the need to talk with a professional, such as a therapist, psychiatrists, or psychologist?,” “Did you have a talk with a professional?,” “Do you still feel the need for professional help?,” “Did you go to professional aid before COVID-19?,” and “Were you satisfied with the aid you received?”

The following questions were multiple-choice questions, where multiple answers were possible. “Why did you want to talk with a professional?” was answerable with statements on emotional problems (“I felt lonely,” “I felt sad, angry, stressed, anxious,” “I had no pleasure anymore in doing things,” “I had suicidal thoughts,” and “I wasn’t feeling good about myself”), situational problems (“I had problems at home,” “I had problems at school,” and “I was being bullied”), and behavioral problems (“I was hurting myself (e.g., automutilation)” and “I had/have an eating disorder”). Other options were “I just wanted to talk with someone” and “Other,” which was an open-text field. “How did you find this professional?” was answerable with “Parents,” “Brothers/sisters,” “Family,” “Friends,” “Girl/boyfriend,” “Student counseling,” “Online,” “Youth helplines (e.g., Awel),” and “I went to professional aid before COVID.”

For those who did not need professional aid: “If you needed to find professional help, would you know where to find it?” was answerable with “I would not know where to find it,” “I would ask help to family/friends,” “I would ask help in school,” “I already know someone that provides professional aid,” “I would seek help online,” and “I would ask aid through online-aid (e.g., Awel, Tele-onthaal).” Tele-onthaal is an organization anyone can call or contact for any subject.

For those who did not talk to a professional: “Why did you not seek out help?” was answerable with “Fear of the reaction of parents/family,” “Fear of the reaction of friends,” “It was online and I do not have the necessary material to do that,” “It was only online and I did not want to do it like that,” “It was online and I did not have a place at home to do it privately,” “My therapist told me he/she could not do it,” “I thought it was not possible because of the COVID-measures,” “The measures did not allow me to go there physically,” “It was only possible physically, and I did not feel comfortable doing that,” “Too expensive,” “I did not know where to go,” “No time,” and “Other reason,” which was completable as a free-text field.

Perceived stigma was assessed by asking if respondents feared negative reactions from (a) parents/family or (b) friends; an affirmative response to either was counted as stigma.

“Who did you talk to about your problems?” was answerable with “My parents,” “My brother/sister,” “My friends,” “My classmates,” “A teacher,” “Student counseling,” “Youth helplines (e.g., Awel),” “No one,” and “Other,” which was a free-text field.

For those that received aid, but were not satisfied with it: “Why were you not satisfied?,” with answering options “It was only online and I do not like it online,” “It was only online and I had problems with getting on the internet,” “It was too expensive,” “I did not feel understood,” “It was a physical conversation and I did not feel comfortable with this,” “It was too short,” and “Other reason” (open text field).

One question was in the form of a Likert scale. “Was this talk online or offline?” was answerable with “Completely online,” “Mostly online, but sometimes physical,” “Partly online, partly physical,” “Mostly physical, but sometimes online,” and “Completely physical.”

### 2.3 Analysis

Those who were older than 25 years of age were excluded. Similarly, those who did not provide an answer to the question about age were removed. Finally, we excluded all those who did not complete the question about whether or not they needed aid. Descriptive statistics were calculated with Pearson’s chi-squared test when comparing groups. Open-text fields were summarized and reported descriptively.

### 2.4 Ethics

This study was approved by the ethics committee of the VUB/UZ Brussels (B.U.N. 1432021000442). Respondents first got an introduction screen explaining the aims of the study with the assurance that any response given would be processed and presented anonymously. At the end of the survey, respondents received information on how to contact Awel or WAT WAT if they were in need of information or help.

## 3 Results

### 3.1 Demographics

In total, 1,079 people completed the survey. After excluding those of unknown age and without information on the need for professional aid, respectively, 982 respondents remained. As can be seen in Table 1, the large majority of respondents were female (81.4%). The majority were between 10 and 15 years old (66.7%). The largest age category was between 13 and 15 years old (44.8%).

**Table 1:** Characteristics of children and adolescents responding to the online survey of mental health needs and access during COVID-19.

	Total (*n* = 982)	Those who needed professional aid (*n* = 636)			Those who did not need professional aid (*n* = 346)
	*N* (%)	*N* (%)	Those who had professional aid (*n* = 188) (%)	Those who did not receive professional aid (*n* = 448) (%)	*N* (%)
Gender					
Male	157 (16)	84 (13.2)	26 (13.8)	52 (11.6)	73 (21.1)
Female	799 (81.4)	538 (84.6)	159 (84.6)	366 (81.7)	261 (75.4)
Other	11 (1.1)	10 (1.6)	3 (1.6)	6 (1.3)	1 (0.3)
No answer	15 (1.5)	4 (0.6)	0 (0)	3 (0.7)	11 (3.2)
Age					
10–12	215 (21.9)	89 (14)	26 (13.8)	59 (13.2)	126 (36.4)
13–15	440 (44.8)	301 (47.3)	71 (37.8)	221 (49.3)	139 (40.2)
16–17	148 (15.1)	116 (18.2)	29 (15.4)	84 (18.8)	32 (9.2)
18–19	59 (6.0)	42 (6.6)	14 (7.4)	26 (5.8)	17 (4.9)
20–25	120 (12.2)	88 (13.8)	48 (25.5)	37 (8.3)	32 (9.2)
Living situation					
I live with one parent at a time	179 (18.2)	115 (18.1)	30 (16)	78 (17.4)	64 (18.5)
I live in the same house as my parents	695 (70.8)	447 (70.3)	123 (65.4)	314 (70.1)	248 (71.7)
I live with another family other than my parents	11 (1.1)	6 (0.9)	5 (2.7)	1 (0.2)	5 (1.4)
I live in a group	14 (1.4)	12 (1.9)	9 (4.8)	2 (0.4)	2 (0.6)
I live alone	30 (3.1)	18 (2.8)	10 (5.3)	7 (1.6)	12 (3.5)
I live in another way	44 (4.5)	33 (5.2)	10 (5.3)	23 (5.1)	11 (3.2)

### 3.2 Needing professional aid

Among the respondents, 636 (64.8%) reported that they felt a need for PMHAA. Girls generally stated to experience a greater need than boys (84.4 and 13.2%) (*p* < 0.001). The majority of those between 13 and 25 reported needing aid, while this was only a minority among those between 10 and 12 (85.9 and 14%) (*p* < 0.001) (see Table 1). The main reasons for needing PMHAA were not feeling like themselves (66%), lack of pleasure in doing anything (44.7%); feelings of sadness, anger, stress, or anxiety (68.7%); and loneliness (50.3%). In total, 26.3% of respondents reported that they were hurting themselves, and 37.7% stated that they had suicidal thoughts. Issues at home (37.7%) appeared to be another frequently mentioned reason for needing PMHAA. Of 13.5% of those needing professional mental health aid, these issues were for which they were already in treatment before COVID-19 (see Table 2).

**Table 2:** Motivation for wanting PMHAA (*N* = 636).

	*N* (%)
Loneliness	320 (50.3)
Feelings of sadness, anger, stress, or anxiety	437 (68.7)
Lack of pleasure in doing anything	284 (44.7)
Suicidal thoughts	240 (37.7)
Problems at home	240 (37.7)
Problems at school	219 (34.4)
Bullying	64 (10.1)
Self-harm	167 (26.3)
Eating disorder	106 (16.7)
Not feeling like themselves	420 (66.0)
Just wanting to talk to someone	277 (43.6)
Pre-COVID issues	86 (13.5)
Other	75 (11.8)

Of those reporting that they needed a conversation with a professional, 188 (29.6%) had an appointment, with 59 of them already going to a professional before the COVID-19 pandemic (31.4% of 188). Others found aid through student counseling (18.1%) or parents (44.1%) (see Table 3). For 48.9%, the aid they received was entirely in person, with only 12.8% having completed online sessions. The majority were satisfied with this approach, and only 25.5% indicated that they were dissatisfied. The reason for dissatisfaction was varied, with the most frequent answer being that the respondent did not feel understood (4.8%).

**Table 3:** Directed respondents to PMHAA (*N* = 188).

	*N*	% (*n* = 188)	% of <18 years old
Parents	83	44.1	38.8
Brothers/sisters	7	3.7	2.7
Family	9	4.8	3.2
Friends	14	7.4	4.8
Boyfriend/girlfriend	11	5.9	4.3
Someone from school	34	18.1	13.8
Social workers	12	6.4	3.2
Websites	19	10.1	0.5
Online chat channels (youth helplines)	10	5.3	2.1
Already went to the psychologist before COVID-19	59	31.4	16.0
Other	19	10.1	8.5

### 3.3 Needing, but not seeking professional aid

Of the 636 that needed PMHAA, the majority (*n* = 448; 70.4% of 636) reported not having an appointment. Children and adolescents (10–17 years) more often had no professional aid (74.3%) than young adults (18–25 years) (50.4%) (see Table 1). The most frequently mentioned reasons for not having an appointment were fear of negative reactions from parents and family (52.7%) and not knowing where to search for professional aid (38.2%). Stigma was present among 54% respondents, with most of these being females (46.7% of 448 respondents). It was present mostly among minors (48.4%), and those between 13 and 15 years old (30.6%) experienced stigma more than other age groups. (see Table 4). Other reasons included that it was too expensive (12.9%), 5.6% thought it was not possible due to the COVID-19 measures, and 4.9% did not go because of the COVID-19 measures. However, 12.1% indicated that they did not seek out psychological aid because it was only possible through online media.

**Table 4:** Reason for not seeking out PMHAA.

	Total (*n* = 448)	% (*n* = 448)	% of minors (<18)
Fear of the reaction of parents/family	236	52.7	47.3
Fear or reaction from friends	96	21.4	19.9
Stigma	242	54	48.4
Online as a barrier because of the lacking material	3	0.7	0.4
Online as a barrier because of personal preference	54	12.1	5.8
Online as a barrier because of privacy	43	9.6	7.4
Appointments canceled by the therapist	1	0.2	0.0
Thought COVID-19 measures did not allow therapy	25	5.6	4.2
COVID-19 measures did not allow physical aid	22	4.9	3.3
Only physical aid and uncomfortable doing that	21	4.7	3.3
Too expensive	58	12.9	8.3
Not knowing who to go to	171	38.2	33.9
No time	29	6.5	4.5
Other reason	125	27.9	19.0

Those who completed the open text field (27.9%) indicated reasons such as not wanting to overburden their parents (3.1%), fear of making the appointment (3.4%), feeling their issues were not serious enough (1.1%), fear of being mocked by family (1.3%), and long waiting lists (0.9%). To cope with their issues, most talked to friends (33.4%), though a large proportion also did not talk to anyone (38.2%). Of those that needed a professional but eventually did not, 75.2% still needed to have a talk with a professional.

### 3.4 Not needing any professional aid

In total, 346 (35.2%) respondents had no need for PMHAA. We asked them who they would reach out to for help finding professional aid if needed. The majority of respondents indicated family or friends (37% of 346), though 29.2% also would seek help online.

## 4 Discussion

This study investigated the mental health needs of children, adolescents, and young adults during the COVID-19 pandemic. Our findings reveal a substantial prevalence of unmet professional mental health needs among the respondents. Those individuals who sought mental health assistance during the pandemic were mostly those who were already engaged in such services prior to the outbreak, and mostly older participants (+18) sought mental health assistance. Conversely, younger participants (below 18 years old) expressed a desire for professional mental health support but refrained from seeking help for various reasons. The most frequently cited factors were stigma (54%), lack of awareness about available professional aid (38.2%), and financial constraints (12.9%). While some respondents (12.1%) mentioned not needing aid when online psychological assistance was the only option, COVID-19 measures were rarely identified as a direct hindrance to seeking aid. Those who did not seek professional mental healthcare often did not talk to anyone about their mental health issues (38.2%).

This study’s findings align with research conducted in other countries during the COVID-19 pandemic. For instance, a survey encompassing young adults from Canada and France reported that 50.7% expressed a need for mental health services, although a majority did not access them (24). Stigma emerged as the primary barrier to access to professional mental health aid in our study. Despite Belgium having a fairly well-developed mental health system, stigma predominantly hindered minors from reaching out to the system (25, 29, 30). This suggests that the challenges surrounding mental healthcare and youth persistently existed both before and after the onset of COVID-19. However, the pandemic potentially exacerbated the issue by introducing online therapy, which some respondents found problematic. Encouragingly, those who predominantly received mental healthcare online reported comparable levels of satisfaction with the therapy as those who exclusively received in-person therapy.

Among those younger than 18 years old, there is a markedly lower reported need for mental health aid among 10–12-year-olds. This could indicate genuinely lower incidence of psychosocial distress in pre-adolescents, but might also reflect under-recognition of mental health needs in this age group. Thus, this might indicate a need for more mental health literacy. However, it is difficult to make broad conclusions about this finding due to lower survey engagement by younger children.

This study revealed that individuals aged 18 and older, along with those who had established prior contact with therapists, were more successful in seeking out mental health aid. This pattern mirrors findings in studies on adults, indicating that those with pre-existing engagement with mental health experts were better positioned to access professional services during the pandemic compared to individuals who developed mental health concerns amid the COVID-19 crisis (20, 31). This is not surprising, as studies have shown the strong link between the need for professional mental health and the actual seeking of mental health aid, when possible (32).

Another noteworthy challenge identified was respondents’ lack of awareness regarding where to seek mental health aid. This confusion may partly arise from Belgium’s intricate mental health system (33). To address this issue, it might be beneficial to introduce educational modules in schools to inform youth about where to find and how to access professional mental health aid. Nevertheless, the implementation of such initiatives remains unclear due to the scarcity of robust longitudinal studies (25). Despite improving familiarity with the mental health system, financial concerns persist as a substantial barrier, as evident in other studies (34). It is all the more surprising, however, since in general therapy sessions in Belgium are free for the first session (33). It shows that taking away financial barriers is not enough to increase access to mental health aid.

The study’s limitations include its cross-sectional design, precluding causal inferences or follow-up investigations. Moreover, the convenience sampling method used for the online survey hampers its representativeness. For example, girls are overrepresented in the sample. The manner of data collection, using sites that are aimed at helping youth with various problems and questions, may also indicate a bias toward respondents who already wanted aid. Additionally, Belgium, and Flanders in particular, generally have a quite high burden of mental health disorders, making the results in this study probably not directly comparable with other countries (35). Finally, the questionnaire’s simplicity restricted in-depth exploration. Related to that, the concept of “stigma” as operationalized in this study does not fully capture the full complexity of stigma. Nevertheless, the strengths of this study lie in its status as one of the few to assess youth’s access to professional mental health aid during COVID-19, particularly with a substantial sample size and a wide age range.

Future studies should assess further the access of youth to professional mental health aid. Clearly, this is an issue that will not go away with time, as it was already noticed before COVID-19 and during COVID-19. In addition, studies should also look at how youth helplines may aid in addressing the issues that youth encounter. It may be that youth helplines are more low-threshold and thus easier to access for youth, while having the same effect. However, to our knowledge, no such studies exist that have evaluated whether youth helplines can aid in this. Either way, it is clear that solutions have to be sought out to address this clear problem.

## 5 Conclusion

In summary, our study underscores the difficulties faced by individuals aged 10 to 25 in accessing professional mental health support, with the underlying barriers being largely consistent with factors predating the COVID-19 pandemic. We propose public health campaigns aimed at enhancing mental health literacy among youth, providing them with guidance on accessing professional mental health aid, and combating the stigma associated with mental health issues. Furthermore, addressing financial obstacles is crucial. While progress has been made in reducing mental healthcare costs in Belgium in recent years, further steps should be taken (33).

Finally, the large percentage of respondents who did not talk to anyone about their issues is rather surprising. Social support from friends and family remains one of the most important buffers against the development of mental health issues (36, 37). A campaign to reduce mental health stigma can increase the social support people receive, and thus would in fact reduce the number of people who have to seek professional mental health aid, and would lower the threshold of seeking professional mental health aid for those who need it.

## Data Availability

The raw data supporting the conclusions of this article will be made available by the authors, without undue reservation.
